# Integrated care: from policy to implementation – The Singapore Story

**Published:** 2012-09-04

**Authors:** Jason Cheah, Wong Kirk-Chuan, Harold Lim

**Affiliations:** Agency for Integrated Care, Republic of Singapore; Agency for Integrated Care, Republic of Singapore; Agency for Integrated Care, Republic of Singapore

Globally, healthcare systems have been largely centered on acute care characterized by episodic, high-level (often expensive) care with sophisticated medical sub-specialization. There is now consensus globally that there should be a fundamental refocus by healthcare systems towards more care outside of the acute setting so as to deal with the challenges of an aging population and increased prevalence of chronic diseases. Singapore has a resident population of some 5.5 million people and is also one of the fastest aging populations in the world today. This presents tremendous challenges and opportunities for designing and implementing different care delivery models that meet the needs of an aging population.

In 2009, the Agency for Integrated Care in Singapore was formed. The Agency was created to drive care integration efforts nationally and to develop and link primary care and intermediate and long-term care providers (ILTC) with the acute-focused public healthcare system, as well as to empower patients through education and help them to better navigate the healthcare system.

The vision for healthcare delivery in Singapore is to develop integrated Regional Healthcare Systems, which are essentially geographically-defined patient-centric healthcare ecosystems comprising of partners from the primary, acute and community care sectors, working together to deliver comprehensive and holistic healthcare services to improve patient outcomes. Integrated clinical pathways (ICPs) will ensure continuity of care and care coordinators will ensure the patient transitions are seamless and coordinated. The fragmented nature of long-term care today results in frequent readmissions, delayed discharges from hospitals, premature institutionalization and caregiver stress. As such, in Singapore, expansion of home care and other community-based care services aims to create a care continuum which ‘plugs’ the gaps and promotes ‘aging in place’. Integration of care will allow quality care to be provided at the most cost-effective site, by the right persons, at the right time.

Within the Regional Health Systems, new and innovative initiatives to establish networks with the ILTC and primary care providers, develop new community preventive health initiatives and improve transitional care for patients are currently underway. Bridging gaps between tertiary, secondary and primary care settings to integrate services will bring about transformation of our healthcare delivery system to meet the challenges of increasing demand as our population ages. Innovative approaches such as the Singapore Programme for Integrated Care of the Elderly (SPICE), which is modelled after the US-based Programme for All Inclusive Care of the Elderly (PACE), telecare and end-of-life programmes are being tried and tested for their cost-effectiveness and social acceptance by patients and their families.

What we have learnt is that implementing integrated care is in itself, not a purely ‘technical’ challenge, but one which involves a great deal of change in mindsets amongst healthcare professionals and also amongst patients and their families/care givers. New skills such as inter-professional collaboration, negotiations, conflict resolution, engagement of patients and families, and a data-driven practice are required in order to make integrated care ‘work’.

Powerpoint presentation available at http://www.integratedcare.org at congresses – San Marino – programme.

